# Efficacy and Safety of Traditional Chinese Herbal Medicine for Antipsychotic-Related Constipation: A Systematic Review and Meta-Analysis of Randomized Controlled Trials

**DOI:** 10.3389/fpsyt.2021.610171

**Published:** 2021-04-29

**Authors:** Wen-Wang Rao, Juan-Juan Yang, Han Qi, Sha Sha, Wei Zheng, Ling Zhang, Gabor S. Ungvari, Chee H. Ng, Yu-Tao Xiang

**Affiliations:** ^1^Institute of Mental Health, Hebei Mental Health Centre, Baoding, China; ^2^Unit of Psychiatry, Department of Public Health and Medicinal Administration, Faculty of Health Sciences and Institute of Translational Medicine, University of Macau, Macao, China; ^3^Centre for Cognitive and Brain Science, University of Macau, Macao, China; ^4^Department of Psychiatry, Chaohu Hospital of Anhui Medical University, Hefei, China; ^5^Anhui Psychiatric Center of Anhui Medical University, Hefei, China; ^6^The National Clinical Research Center for Mental Disorders and Beijing Key Laboratory of Mental Disorders, Beijing Anding Hospital and the Advanced Innovation Center for Human Brain Protection, Capital Medical University, Beijing, China; ^7^The Affiliated Brain Hospital of Guangzhou Medical University (Guangzhou Huiai Hospital), Guangzhou, China; ^8^Beijing Municipal Key Laboratory of Clinical Epidemiology, Department of Epidemiology and Health Statistics, School of Public Health, Capital Medical University, Beijing, China; ^9^The University of Notre Dame Australia, Fremantle, WA, Australia; ^10^Division of Psychiatry, School of Medicine, University of Western Australia, Perth, WA, Australia; ^11^Department of Psychiatry, The Melbourne Clinic and St. Vincent's Hospital, University of Melbourne, Richmond, VIC, Australia

**Keywords:** meta-analysis, randomized controlled study, constipation, traditional Chinese medicine, antipsychotic

## Abstract

**Background:** Constipation is a common but often ignored side effect of antipsychotic treatment, although it is associated with adverse outcomes. The results of the efficacy and safety of traditional Chinese herbal medicine (TCM) in treating constipation are mixed across studies. This is a systematic review and meta-analysis of randomized controlled trials (RCTs) of the efficacy and safety of TCM compared to Western medicine (WM) in treating antipsychotic-related constipation.

**Methods:** Major international electronic (PubMed, EMBASE, Cochrane Library, and Web of Science) and Chinese (Wanfang, WeiPu VIP, SinoMed, and CNKI) databases were searched from their inception to November 29, 2020. Meta-analysis was performed using the random-effects model.

**Results:** Thirty RCTs with 52 arms covering 2,570 patients in the TCM group and 2,511 patients in the WM group were included. Compared with WM, TCM alone was superior regarding the moderate response rate [risk ratio (RR) = 1.165; 95% confidence interval (CI): 1.096–1.238; *P* < 0.001], marked response rate (RR = 1.437; 95% CI: 1.267–1.692; *P* < 0.001), and remission rate (RR = 1.376; 95% CI: 1.180–1.606; *P* < 0.001) for constipation, while it was significantly associated with lower risk of rash (RR = 0.081; 95% CI: 0.019–0.342; *P* = 0.001). For the moderate response rate, meta-regression analyses revealed that publication year (β = −0.007, *P* = 0.0007) and Jadad score (β = 0.067, *P* < 0.001) significantly moderated the results. For the remission rate, subgroup and meta-regression analyses revealed that the geographical region (*P* = 0.003), inpatient status (*P* = 0.035), and trial duration (β = 0.009, *P* = 0.013) significantly moderated the results.

**Conclusions:** The efficacy of TCM for antipsychotic-related constipation appeared to be greater compared to WM, while certain side effects of TCM, such as rash, were less frequent.

## Introduction

Constipation is a common side effect of antipsychotics with a prevalence rate between 28.1 and 36.3% (1–3) and is associated with a range of severe consequences, such as paralytic ileus, bowel ischemia, sepsis, intestinal perforation, and even pre-mature mortality (4, 5). The occurrence of constipation in psychiatric patients may be associated with a decrease in gastrointestinal hypomotility due to peripheral muscarinic anticholinergic activity (6, 7). For instance, certain antipsychotics, such as clozapine, quetiapine, and olanzapine (8), have strong affinity to muscarinic cholinergic receptors, which could increase peripheral muscarinic anticholinergic activity (9, 10) and may result in constipation.

Commonly used Western medicine (WM) for constipation, including fiber supplements and laxatives, could cause side effects including nausea, vomiting, diarrhea, and even severe adverse events in certain special populations such as those with renal insufficiency (11, 12). Traditional Chinese herbal medicine (TCM) is commonly prescribed in treating and preventing constipation in clinical practice, particularly in Asian countries such as China (13–15), with good evidence found in some high-quality studies (16–20).

To date, findings on the efficacy and safety of TCM for antipsychotic-related constipation compared with WM have been inconsistent. Recent reviews (21, 22) summarized the efficacy of TCM for antipsychotic-related constipation but only included publications in English databases, even though most relevant studies were only published in Chinese language journals. Consequently, only two studies conducted in China were included; one study (23) focused on physical therapy of traditional Chinese Medicine (e.g., acupuncture and Tuina) and the other focused on the use of 250 ml of 10% mannitol with 2 g of Rhubarb-soda plus 0.8 g of Phenolphthalein Tablets (24). This gave us the impetus to conduct this systematic review and meta-analysis of randomized controlled trials (RCTs) of the efficacy and safety TCM and WM in treating antipsychotic-related constipation.

## Materials and Methods

This meta-analysis was registered in PROSPERO (CRD42020168832) and was performed in accordance with the Preferred Reporting Items for Systematic Reviews and Meta-Analyses (PRISMA) statement.

### Eligibility Criteria and Outcome Measures

According to the PICOS acronym (25), the inclusion criteria were as follows: Participants (P): patients with constipation caused by antipsychotic medications. Intervention (I): TCM alone. Comparison (C): WM alone or concurrent use of two or more WMs. Outcomes (O): efficacy and safety of TCM. Study design (S): RCTs. Exclusion criteria included (a) severe physical comorbidities and (b) receiving physiotherapy alone or a combination of physiotherapy plus TCM for constipation. Primary outcome included three efficacy measures: moderate response rate, marked response rate, and remission rate. Secondary outcomes included treatment adherence and adverse drug reactions (ADRs), such as nausea, vomiting, and rash.

### Search Strategy and Study Selection

Literature search in both international (PubMed, EMBASE, Cochrane Library, and Web of Science) and Chinese (Wanfang, WeiPu VIP, SinoMed, and CNKI) databases from inception to October 30, 2019, were independently conducted by two researchers (WWR and JJY), using both subject and free terms of the following search terms: “Constipation [MeSH],” “Medicine, Chinese Traditional [MeSH],” and “Randomized Controlled Trial [MeSH]” (Supplementary Table 2). An updated search to November 29, 2020, was also performed.

The same two researchers (WWR and JJY) independently screened titles and abstracts and then read full texts of relevant publications for eligibility. Any discrepancy was discussed with a third researcher (ZW). In addition, the reference lists of relevant reviews and previous meta-analysis (21, 22) were searched manually for additional studies.

### Data Extraction

A pre-designed Excel data collection sheet was used to independently extract relevant data by two researchers (WWR and JJY). The following study and participant characteristics were extracted: the first author, year of publication and survey, sample size, type of medications, mean age of participants, proportion of males, and diagnostic criteria of psychiatric disorders and constipation. Any disagreement was resolved by consensus.

### Quality Assessment and Evidence Level

The two researchers (WWR and JJY) independently assessed study quality using both the Jadad scale (0–5 points) (26) and Cochrane risk of bias tool (27). Studies with a Jadad total score of 3 or higher were considered as “high quality;” otherwise, they were considered as “low quality.” The Grading of Recommendations Assessment, Development, and Evaluation (GRADE) methodology was used to evaluate evidence level of primary and secondary outcomes (i.e., very low, low, moderate, or high) (28).

### Statistical Analyses

Due to different sample sizes, types and doses of antipsychotic medications, and demographic characteristics between studies, the random-effects model was used to synthesize outcome data, with risk ratio (RRs) and its 95% confidence intervals (CIs) as the effect size. Heterogeneity was assessed using the Cochran's *Q* and *I*^2^ statistic. *I*^2^-values of ≥50% and *P*-value of ≤ 0.10 indicated great heterogeneity across studies. Publication bias was tested using forest plots, Egger's regression test, Begg's rank test, and Duval and Tweedie's trim-and-fill analysis. The sources of heterogeneity between studies on primary outcomes (e.g., moderate/marked response and remission rates of constipation) were examined by subgroup analyses for categorical variables [e.g., diagnostic criteria for psychiatry: Chinese Mental Disorder Classification and Diagnosis, Third Edition (CCMD-3) vs. Chinese Mental Disorder Classification and Diagnosis, Second Edition (CCMD-2)/Chinese Mental Disorder Classification and Diagnosis, Second Edition, Revised (CCMD-2-R) vs. International Classification of Diseases, Tenth Edition (ICD-10), geographic region (east vs. middle vs. west), analysis method (intent to treat vs. per-protocol), and inpatient group (Yes vs. Mix)] and meta-regression analyses for continuous variables (e.g., publication year, trial duration, Jadad total score, and overall sample size). Sensitivity analysis was carried out to identify outlying studies. All statistical analyses were performed using Comprehensive Meta Analysis (version 2.0; Biostat), with a significance level of 0.05 (two-sided).

## Results

### Literature Search and Study Characteristics

**Figure 1:**
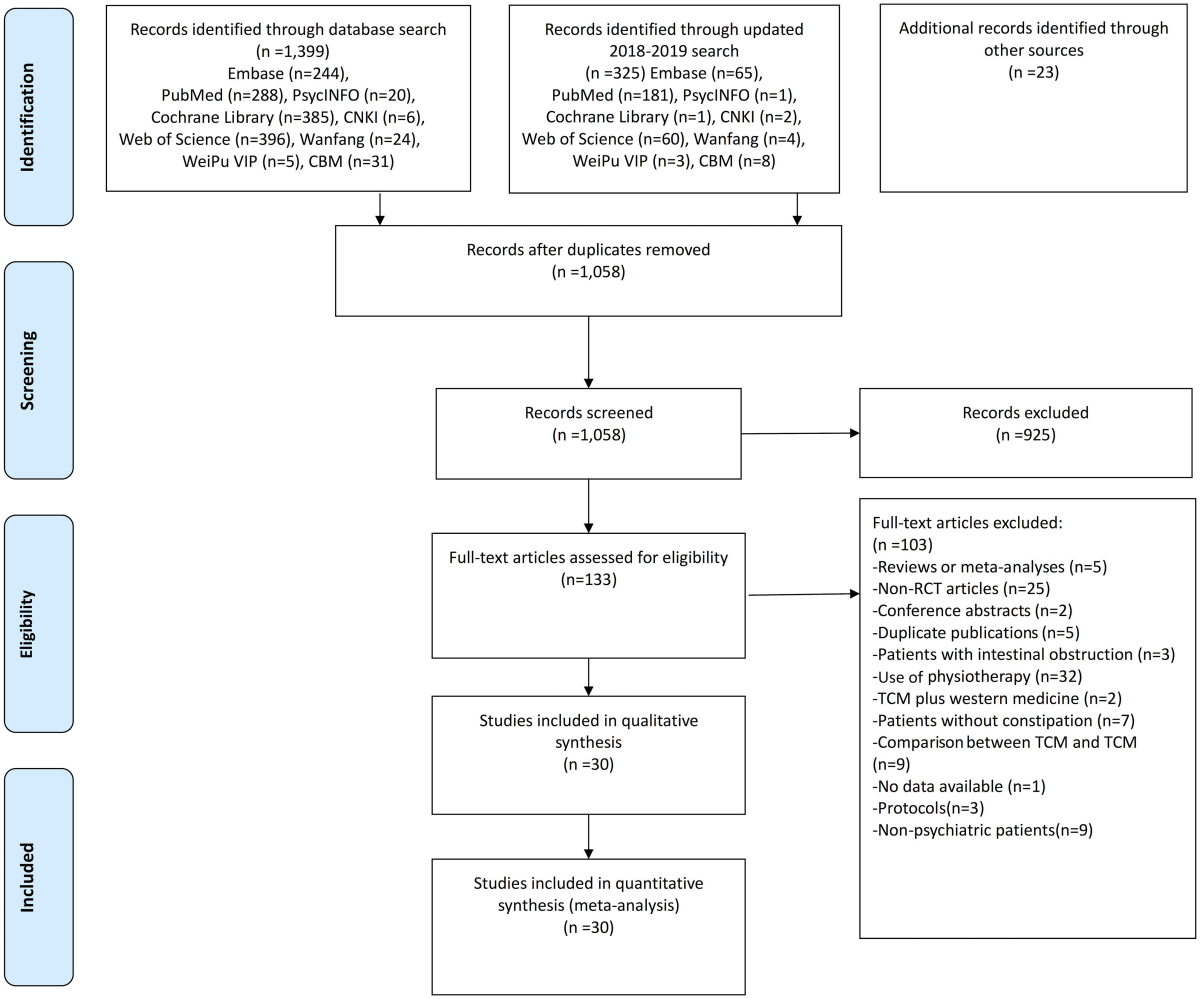
The flow charts of included studies.

A total of 1,725 articles were initially identified. After screening the titles and abstracts, 133 articles were retrieved for full-text review. Finally, 30 studies with 52 arms (2,570 patients in the TCM group and 2,511 patients in the WM group) were included for meta-analyses (Figure 1).

Included studies were published from 1993 to 2020. All studies were conducted in China: 19 studies were conducted in the eastern region, 8 in the central region, and 3 in the western region of China. Sixteen studies used the CCMD-3; two used the CCMD-2; one used the CCMD-2-R; one used the ICD-10; and ten studies did not report diagnostic criteria. The sample size ranged from 60 to 328, and mean age ranged between 28.08 and 69.85 years. Study duration ranged from 0.42 to 28 days (Table 1).

Table 1Characteristics of studies included in this meta-analysis.

**Table:** 

**No**.	**First author**	**Publication** ** year**	**Survey** ** year**	**Total** ** sample** ** size**	**Age** ** (Mean ± SD)**	**Age** ** range**	**Male** ** (%)**	**ACT**	**Province**	**Region**	**Inpatients**	**Analysis**	**Diagnostic** ** criteria**	**Type of disorder**	**Type of medication**	**Diagnostic criteria for constipation**	**Randomization**	**Blinding**	**Withdrawal and dropouts**	**Total score of Jadad**	**References**
1	Zhao et al.	1993	1991–1992	180	38.6 ± 11.64	18–69	144 (80)	(117.3 ± 6.84) h	Shandong	E	Yes	ITT	NR	SCH, AD, ND, and PMD	CL, CH, PE, HA, TF, and others	At least 4 days without stool	2	2	0	4	(29)
2	Ding	1998	1996	174	NR	17–60	144 (82.8)	NR	Jiangsu	E	Yes	ITT	CC-MD-2	SCH, AD, and others	CL and others	72 h without stool	1	0	0	1	(30)
3	Wang et al.	1998	NR	181	36.19 ± 8.80	18–52	117 (64.6)	(4.62 ± 0.60) d	Shanxi	M	Yes	ITT	NR	SCH	CL	Lasting 4 days or more with no stool	1	0	0	1	(31)
4	Liu et al.	2001	NR	60	38.05 ± 7.89	NR	44 (73.33)	NR	Shaanxi	W	Yes	ITT	NR	SCH	PA	Criterion I	1	0	0	1	(32)
5	Hu et al.	2002	2000–2001	90	67.4 ± 12.6	18–87	48 (53.3)	NR	Guangdong	E	Yes	ITT	NR	NR	NR	More than 3 days with no stool	1	0	0	1	(33)
6	Li	2003	2002–2003	261	NR	17–60	216 (82.8)	NR	Beijing	E	Yes	ITT	CC-MD-2	SCH, AD, and others	CL and others	72 h without stool	1	0	0	1	(34)
7	Zhang	2003	2001	60	38.3 ± 11.77	NR	27 (45.0)	(5.39 ± 2.85) d	Henan	M	Yes	ITT	NR	NR	NR	Lasting 3 days with no stool	1	0	0	1	(35)
8	Li et al.	2005	1999–2003	97	28.73 ± NR	17–57	56 (57.7)	NR	Guizhou	W	Mix	ITT	CC-MD-3	SCH, MA, RP, and others	CH	Criterion F	1	0	0	1	(36)
9	Li et al.	2005	2003–2004	90	41.3 ± 17.3	18–72	90 (100)	NR	Shandong	E	Yes	ITT	CC-MD-2-R	NR	NR	Criterion A	1	0	0	1	(37)
10	Sheng et al.	2006	2005–2006	118	32.6 ± 3.2	16–56	72 (61.0)	NR	Anhui	M	Yes	ITT	CC-MD-3	SCH, DP, and others	CL, CH, SU, PE, and others	At least 3 days without stool	0	0	0	0	(38)
11	Meng et al.	2007	2004	328	28.47 ± 10.33	NR	229 (69.8)	NR	Shandong	E	Yes	ITT	NR	NR	NR	Criterion G	1	0	0	1	(39)
12	Wang et al.	2007	2002–2006	120	38.2 ± 15.3	16–64	87 (72.5)	NR	Hebei	E	Yes	ITT	CC-MD-3	SCH, AD, and others	NR	Criterion E	1	0	0	1	(40)
13	Du et al.	2008	2006–2007	115	28.8 ± 13.1	18–65	64 (55.7)	NR	Shanghai	E	Yes	ITT	CC-MD-3	SCH	CL, CH, SU, RI, and others	Criterion B	1	0	0	1	(41)
14	Han et al.	2008	2007–2008	150	39.2 ± 1.66	20–72	126 (84.0)	(115.2 ± 6.34) h	Shandong	E	Yes	ITT	CC-MD-3	SCH, AD, ND, and PMD	CL, CH, PE, HA, SU, RI, and others	More than 4 days with no stool	2	2	0	4	(42)
15	Lin et al.	2008	2007–2008	134	33.83 ± NR	15–58	87 (64.9)	NR	Guangdong	E	Mix	ITT	CC-MD-3	SCH, AD, and others	CH, RI, CL, PE, HA, and others	Criterion H	0	0	0	0	(43)
16	Xie et al.	2008	2007	96	NR	16–59	55 (57.3)	NR	Guangdong	E	Yes	ITT	CC-MD-3	SCH, MD, and SRD	CL, PE, HA, and SU	NR	0	0	0	0	(44)
17	Jiang	2009	2008–2009	87	28.08 ± 12.68	19–52	42 (48.3)	NR	Jiangxi	M	Yes	ITT	CC-MD-3	SCH	CL, CH, OL, and QF	Criterion C	1	0	0	1	(45)
18	Liu et al.	2010	2007	305	31.97 ± 10.29	NR	175 (57.4)	NR	Tianjin	E	Yes	ITT	NR	NR	NR	Criterion G	1	0	0	1	(46)
19	Li	2011	2008–2010	76	39.82 ± 11.00	NR	76 (100.0)	(5.33 ± 1.30) d	Henan	M	Yes	ITT	NR	SCH	CL	Lasting 3 days with no stool	1	0	0	1	(47)
20	Pan et al.	2012	2006–2007	80	33.15 ± 15.38	16–60	39 (48.8)	NR	Henan	M	Yes	ITT	CC-MD-3	SCH, AD, and others	CL, CH, SU, CLO, AM, and others	Lasting 3 days with no stool	1	0	0	1	(48)
21	Wang et al.	2013	NR	87	NR	17–60	NR	NR	Shandong	E	Yes	ITT	CC-MD-3	SCH, AD, and others	CL, RI	72 h without stool	1	0	0	1	(49)
22	Chen et al.	2014	2012–2013	258	48 ± 5	27–65	117 (45.4)	NR	Jiangsu	E	Yes	ITT	CC-MD-3	SCH, AD, and SAP	CL, RI, and others	72 h without self-defecation	1	0	0	1	(50)
23	Tian et al.	2014	2010–2011	119	69.85 ± 9.65	60–80	0 (0.0)	NR	Beijing	E	Yes	ITT	NR	SCH, ALD, VD, and DP	NR	Criterion J	1	0	0	1	(51)
24	Han	2015	2011–2013	100	NR	18–65*	54* (54.0)	NR	Tianjin	E	Yes	PP	CC-MD-3	SCH	NR	Criterion D	2	0	1	3	(52)
25	Ye et al.	2016	2015	192	NR	16–60	110 (57.3)	NR	Zhejiang	E	Yes	ITT	CC-MD-3	SCH and MD	CL, OL, QF, and RI	Criterion E	0	0	0	0	(53)
26	Zhao et al.	2016	2008–2009	120	49 ± NR	17–86	61 (50.8)	NR	Auhui	M	Yes	ITT	NR	NR	NR	More than 3 days with on stool	1	0	0	1	(54)
27	Tang	2018	2015–2017	80	41.61 ± 11.13	20–60	47 (58.8)	(4.36 ± 1.25) d	Hubei	M	NR	ITT	CC-MD-3	NR	CL	Criterion G	0	0	0	0	(55)
28	Wang et al.	2019	2015–2017	100	38.6 ± 3.2	18–60	NR	NR	Guangdong	E	Yes	PP	ICD-10	SCH	NR	Lasting 3 days without stool + Criterion D	1	0	1	2	(56)
29	Zhu	2019	2017–2018	120	48.1 ± 4.87	25–68	76 (63.3)	NR	Zhejiang	E	Yes	ITT	CC-MD-3	SCH, AD	NR	Lasting 3 days without stool	2	0	0	2	(57)
30	Wu et al.	2020	2018–2019	70	38.85 ± 2.15	22–56	41 (58.6)	NR	Shanxi	W	Yes	ITT	CC-MD-3	SCH	NR	NR	2	0	0	2	(58)

**Table:** 

**Order** ** of arms**	***N***	**Age** ** (Mean** **±** **SD)**	**Age** ** range**	**Male** ** (%)**	**ACT**	**Trial duration** ** (days)**	**TCM**						**WM**					
							***N***	**Age** ** (Mean** **±** **SD)**	**Age ** ** range**	**Male (%)**	**ACT**	**Name**	***N***	**Age** ** (Mean** **±** **SD)**	**Age** ** range**	**Male (%)**	**ACT**	**Name**
1	120	NR	NR	NR	NR	1	60	NR	NR	NR	NR	Senna	60	NR	NR	NR	NR	Phenolphthalein
2	120	NR	NR	NR	NR	1	60	NR	NR	NR	NR	Rhei Radix Et Rhizoma	60	NR	NR	NR	NR	Phenolphthalein
1	95	NR	NR	NR	NR	23.38 (average)	51	NR	NR	NR	NR	Senna Mixture	44	NR	NR	NR	NR	Vitamin B1
2	84	NR	NR	NR	NR	22.94 (average)	51	NR	NR	NR	NR	Senna mixture	33	NR	NR	NR	NR	1.5% Saline
3	97	NR	NR	NR	NR	23.19 (average)	51	NR	NR	NR	NR	Senna Mixture	46	NR	NR	NR	NR	Glycerine Enema/0.2% Soapsuds Enema
1	181	36.19 ± 8.80	18–52	117 (64.6)	(4.62 + 0.60) d	1	89	35.54 ± 8.63	18-47	56 (62.9)	(4.65 ± 0.66) d	Senna	92	36.82 ± 8.92	20–52	61 (66.3)	(4.59 ± 0.54) d	Phenolphthalein
1	60	38.05 ± 7.89	NR	44 (73.33)	NR	7	30	38.3 ± 8.2	NR	23 (76.7)	NR	Yu Zhu Shu Tong	30	37.8 ± 7.7	NR	21 (70.0)	NR	Phenolphthalein
1	60	NR	NR	NR	NR	1	30	NR	NR	NR	NR	Senna	30	NR	NR	NR	NR	Phenolphthalein and Glycerine Enema
2	60	NR	NR	NR	NR	1	30	NR	NR	NR	NR	Senna	30	NR	NR	NR	NR	Phenolphthalein
1	117	NR	NR	NR	NR	25.14 (average)	66	NR	NR	NR	NR	Apricot seed and Linum formula	51	NR	NR	NR	NR	1.5% Saline
2	134	NR	NR	NR	NR	25.00 (average)	66	NR	NR	NR	NR	Apricot seed and Linum formula	68	NR	NR	NR	NR	Glycerine Enema/0.2% Soapsuds Enema
3	127	NR	NR	NR	NR	25.94 (average)	76	NR	NR	NR	NR	Senna mixture	51	NR	NR	NR	NR	1.5% Saline
4	144	NR	NR	NR	NR	25.72 (average)	76	NR	NR	NR	NR	Senna Mixture	68	NR	NR	NR	NR	Glycerine Enema/0.2% Soapsuds Enema
1	60	38.3 ± 11.77	NR	27 (45.0)	(5.39 ± 2.85) d	28	30	39.8 ± 11.1	NR	14 (46.7)	(5.32 ± 3.12) d	Qi Rong Run Chang oral liquid	30	36.8 ± 12.4	NR	13 (43.3)	(5.46 ± 2.61) d	Phenolphthalein
1	97	28.73 ± NR	17–57	56 (57.7)	NR	5	52	28.5 ± NR	18–55	29 (55.8)	NR	Peony and Licorice combination	45	29 ± NR	17–57	27 (60.0)	NR	Phenolphthalein
1	60	NR	NR	60 (100)	NR	1	30	NR	NR	NR	NR	Rhubarb and Mirabilite and Magnolia Officinalis Rehd et Wils formula	30	NR	NR	NR	NR	Phenolphthalein and Glycerine Enema
2	60	NR	NR	60 (100)	NR	1	30	NR	NR	NR	NR	Senna	30	NR	NR	NR	NR	Phenolphthalein and Glycerine Enema
1	118	32.6 ± 3.2	16–56	72 (61.0)	NR	0.42	58	NR	NR	NR	NR	Senna	60	NR	NR	NR	NR	10% Mannitol
1	328	28.47 ± 10.33	NR	229 (69.8)	NR	1	165	NR	NR	NR	NR	Senna	163	NR	NR	NR	NR	Phenolphthalein
1	80	NR	NR	NR	NR	3	40	NR	NR	NR	NR	Tongfu Qingyu decoction	40	NR	NR	NR	NR	Phenolphthalein
2	80	NR	NR	NR	NR	3	40	NR	NR	NR	NR	Senna	40	NR	NR	NR	NR	Phenolphthalein
1	87	29.10 ± 13.20	NR	46 (52.9)	NR	28	38	28.87 ± 13.81	NR	22 (57.9)	NR	Constipation-relief Capsule	49	29.27 ± 12.85	NR	24 (49.0)	NR	Phenolphthalein
2	77	28.80 ± 12.77	NR	42 (54.6)	NR	28	28	27.97 ± 12.81	NR	18 (64.3)	NR	Angelica and Rhubarb Combination	49	29.27 ± 12.85	NR	24 (49.0)	NR	Phenolphthalein
1	100	NR	NR	NR	NR	1	50	NR	NR	NR	NR	Senna	50	NR	NR	NR	NR	Phenolphthalein
2	100	NR	NR	NR	NR	1	50	NR	NR	NR	NR	Rhubarb	50	NR	NR	NR	NR	Phenolphthalein
1	134	33.83 ± NR	15–58	87 (64.9)	NR	7	68	34.34 ± NR	15–58	45 (66.2)	NR	Mazi Ren Wan	66	33.42 ± NR	15–56	42 (63.6)	NR	Phenolphthalein
1	64	NR	18–57	37 (57.8)	NR	1	32	NR	18–57	19 (59.4)	NR	Senna	32	NR	16–56	18 (56.3)	NR	20% Mannitol
2	64	NR	18–59	37 (57.8)	NR	1	32	NR	18–57	19 (59.4)	NR	Senna	32	NR	19–59	18 (56.3)	NR	Glycerine Enema
1	87	28.08 ± 12.68	19–52	42 (48.3)	NR	10	41	27.96 ± 12.75	NR	20 (48.8)	NR	Peony and Licorice combination	46	28.19 ± 12.77	NR	22 (47.8)	NR	Phenolphthalein
1	305	31.97 ± 10.29	NR	175 (57.4)	NR	NR	163	31.59 ± 10.12	NR	97 (59.5)	NR	Rheum Glycyrrhiza decoction	142	32.40 ± 10.51	NR	78 (54.9)	NR	Phenolphthalein
1	76	39.82 ± 11.00	NR	76 (100)	(5.33 ± 1.30) d	28	38	41.39 ± 10.47	NR	NR	(5.39 ± 1.22) d	Maren Runchang Wan	38	38.25 ± 11.43	NR	NR	(5.27 ± 1.39) d	Glycerine Enema/0.2% Soapsuds Enema
1	80	33.15 ± 15.38	16-60	39 (48.8)	NR	28	40	32.6 ± 16.2	18–60	18 (45.0)	NR	Tongbianling	40	33.7 ± 14.7	16–59	21 (52.5)	NR	Blank control
1	39	NR	NR	NR	NR	21	22	NR	NR	NR	NR	Ma Ren Wan	17	NR	NR	NR	NR	Saline
2	43	NR	NR	NR	NR	21	26	NR	NR	NR	NR	Senna	17	NR	NR	NR	NR	Saline
3	44	NR	NR	NR	NR	21	22	NR	NR	NR	NR	Ma Ren Wan	22	NR	NR	NR	NR	Glycerine Enema/Soapsuds Enema
4	48	NR	NR	NR	NR	21	26	NR	NR	NR	NR	Senna	22	NR	NR	NR	NR	Glycerine Enema/Soapsuds Enema
1	123	NR	NR	NR	NR	NR	57	NR	NR	NR	NR	Senna	66	NR	NR	NR	NR	Lactulose
2	128	NR	NR	NR	NR	NR	57	NR	NR	NR	NR	Senna	71	NR	NR	NR	NR	Phenolphthalein
3	121	NR	NR	NR	NR	NR	57	NR	NR	NR	NR	Senna	64	NR	NR	NR	NR	Glycerine Enema/0.2% Soapsuds Enema
1	119	69.85 ± 9.65	60–80	0 (0.0)	NR	28	60	69.3 ± 10.70	60–78	0 (0.0)	NR	Honeyed glycyrrhiza compound decoction	59	70.4 ± 8.5	61–80	0 (0.0)	NR	Glycerine Enema
1	98	NR	18–65*	54* (54.0)	NR	28	49	NR	18–65*	28* (56.0)	NR	Chinese medicine laxative capsule	49	NR	18–61*	26* (52.0)	NR	Phenolphthalein
1	128	NR	16–59	78 (60.9)	NR	3	64	NR	16–57	40 (62.5)	NR	Maren Ruan Capsule	64	NR	19–59	38 (59.4)	NR	Phenolphthalein
2	128	NR	17–59	74 (57.8)	NR	3	64	NR	17–57	36 (56.3)	NR	Senna	64	NR	19–59	38 (59.4)	NR	Phenolphthalein
1	60	NR	NR	NR	NR	0.5	30	NR	NR	NR	NR	Senna	30	NR	NR	NR	NR	Phenolphthalein
2	60	NR	NR	NR	NR	0.5	30	NR	NR	NR	NR	Senna	30	NR	NR	NR	NR	Retention enema with Glycerine Enema
3	60	NR	NR	NR	NR	0.5	30	NR	NR	NR	NR	Senna	30	NR	NR	NR	NR	Glycerine Enema
1	80	41.61 ± 11.13	20–60	47 (58.8)	(4.36 ± 1.25) d	NR	40	41.77 ± 11.34	20–60	32 (80.0)	(4.38 ± 1.25)d	Senna	40	41.45 ± 11.05	21–59	23 (57.5)	(4.33 ± 1.27) d	Phenolphthalein
1	96	38.6 ± 3.2	18–60	NR	NR	28	50	NR	NR	NR	NR	Maren Ruan Capsule	46	NR	NR	NR	NR	Phenolphthalein
1	60	48.15 ± 5.01	25–67	41 (68.3)	NR	NR	30	48.1 ± 5.0	25–67	22 (73.3)	NR	Senna	30	48.2 ± 5.1	26–66	19 (63.3)	NR	Lactulose
2	60	47.95 ± 4.91	25–68	40 (66.7)	NR	NR	30	48.1 ± 5.0	25–67	22 (73.3)	NR	Senna	30	47.8 ± 4.9	26–68	18 (60.0)	NR	Phenolphthalein
3	60	48.2 ± 4.80	25–67	39 (65.0)	NR	NR	30	48.1 ± 5.0	25–67	22 (73.3)	NR	Senna	30	48.3 ± 4.68	25–66	17 (56.7)	NR	Glycerine Enema/0.2% Soapsuds Enema
1	70	38.85 ± 2.15	22–56	41 (58.6)	NR	14	35	38.6 ± 2.2	24–56	21 (60.0)	NR	Peony and Licorice combination	35	39.1 ± 2.1	22–55	20 (57.1)	NR	Phenolphthalein

*Including patients with dropout.

*ACT, Average constipation time; h, Hour; d, Day; TCM, Traditional Chinese medicine; WM, Western medicine; CCMD-2, Chinese Mental Disorder Classification and Diagnosis, Second Edition; CCMD-2-R, Chinese Mental Disorder Classification and Diagnosis, Second Edition, Revised; CCMD-3, Chinese Mental Disorder Classification and Diagnosis, Third Edition; ICD-10, International Classification of diseases, Tenth Edition; Criterion A, Patient with abdominal distension, loss of appetite, difficulty in defecation, and no stool discharge for more than 3 days; Criterion B, One of three symptoms (decreasing times of fecal discharge or dry stool or difficult defecation) and a sign cluster (abdomen fullness and discomfort, palpable cord-like mass, dizziness, headache, short urination, dry mouth, bitter mouth, fatigue, irritability, etc.) due to the accumulation of belly stool (59); Criterion C, Patients with difficult fecal discharge, prolonging defecation time, only defecates once or has a feeling of defecation but cannot defecate in 4–6 days; Criterion D, Rome terion D difficult fe on Functional Constipation; Criterion E, diagnostic criteria from Thompson et al. (60); Criterion F, Diagnostic criteria for constipation with Yin deficiency syndrome (61); Criterion G, Lasting 3 days with no stool; dry stool; laborious defecation; Criterion H, Constipation severity criteria (level 0: without constipation and defecation one time in 1–2 days with soft stool; level 1: defecation one time in 2–3 days after medication and stiff stool into strips with difficulty in defecation; level 2: defecation one time in 3–4 days after medication and stiff stool into granular lumpy with difficulty in defecation; level 3: defecation one time in more than 5 days after medication, and lumpy stool with difficulty in defecation by yourself, even defecation by external forces); Criterion I, difficulty in defecation, no stool discharge for more than 3 days and change of defecation habits; Criterion J, Guideline for the diagnosis and treatment of chronic constipation (62); ITT, Intention to treat analysis; PP, per-protocol analysis; NR, Not Reported; SD, Standard deviation; SCH, Schizophrenia; AD, Affective disorders; DP, Depression; MD, Mood disorders; SRD, Stress-related disorders; ND, Neurotic disorders; PMD, Psychogenic mental disorders, Ma, Mania; RP, Reactive psychosis; SAP, Schizo-affective psychosis; ALD, Alzheimer's disease; VD, Vascular dementia; CL, Clozapine; CH, Chlorpromazine; SU, Sulpiride; PE, Perphenazine; RI, Risperidone; HA, Haloperidol; OL, Olanzapine; QF, Quetiapine fumarate; TF, Trifluoperazine; PA, Phenothiazine antipsychotics; CLO, Clomipramine; AM, Amitriptyline*.

### Assessment Quality and Outcome Evidence

The mean Jadad scores of the 30 studies ranged from 0 to 4 with a median of 1; of them, 3 were considered as “high quality” (Table 1). Non-blinded assessment and omission of reported dropout were the major reasons for low quality. For the assessment of Cochrane risk of bias, five RCTs mentioned “randomization” in detail (i.e., low risk), and five RCTs used randomization with incorrect methods (i.e., high risk). In addition, no RCT described allocation concealment; therefore, the biases were unclear. Two RCTs mentioned “blinding” (Supplementary Figure 1). The overall quality of the 13 meta-analyzable outcomes was rated as “moderate” (15.4%, 2/13) and “high” (3.03%, 1/13) according to the GRADE approach (Supplementary Table 1).

### Systematic Review and Meta-Analysis

#### Response Rate

Traditional Chinese herbal medicine alone had significant advantages in terms of the moderate response rate (RR = 1.165; 95% CI: 1.096–1.238, *P* < 0.001, *I*^2^ = 77.17%, Table 2, Supplementary Figure 2 and Supplementary Table 3), marked response rate (RR = 1.437; 95% CI: 1.267–1.692, *P* < 0.001, *I*^2^ = 81.40%, Table 2, Supplementary Figure 3 and Supplementary Table 3), and remission rate (RR = 1.376; 95% CI: 1.180–1.606, *P* < 0.001, *I*^2^ = 78.88, Table 2, Supplementary Figure 4 and Supplementary Table 3) compared to WM. In contrast, no significant difference was found regarding the onset of response after treatment between TCM alone and WM groups (SMD = −0.142; 95% CI: −0.783–0.499; *P* = 0.664; *I*^2^ = 91.45, Table 2).

**Table 2:** Primary and secondary outcomes of traditional Chinese medicine for constipation.

**Variables**	**Number of studies**	**Case (*n*)**	**Control (*n*)**	**RRs/SMD (95% CI)**	***I^**2**^* (%)**	***Q* (*P*)**	***P***	**Classic**	**Begg**	**Egger**	**Trim and fill**		
								**fail-safe *N***	**(*P*)**	**(*P*)**	**(adjusted value, RRs, 95% CI)**		
**Clinical efficacy:**													
Moderate response rate	52	2,570	2,511	1.165 (1.096–1.238)	77.17	210.27 (<0.001)	** <0.001**	933	0.005	<0.001	8	1.100	1.030–1.174
Marked response rate	44	2,167	2,105	1.437 (1.267–1.692)	81.40	231.16 (<0.001)	** <0.001**	1,126	<0.001	<0.001	10	1.219	1.067–1.392
Remission rate	31	1,641	1,581	1.376 (1.180–1.606)	78.88	142.02 (<0.001)	** <0.001**	368	<0.001	<0.001	2	1.440	1.231–1.685
Time of onset	5	276	216	−0.142 (−0.783–0.499)	91.45	46.78 (<0.001)	0.664	0	0.624	0.653	1	−0.028	−0.592–0.536
**Treatment adherence:**													
Total adherence rate	4	192	192	0.988 (0.785–1.242)	78.18	13.75 (0.003)	0.915	0	0.497	0.872	0	0.988	0.785–1.242
Full adherence rate	4	192	192	0.974 (0.558–1.700)	86.14	21.65 (<0.001)	0.926	0	0.497	0.859	0	0.974	0.558–1.700
Partial adherence rate	4	192	192	1.024 (0.725–1.448)	1.51	3.05 (0.385)	0.891	0	1.000	0.716	0	1.024	0.725–1.448
**Adverse drug reactions:**													
Diarrhea	18	822	733	1.596 (0.976–2.610)	58.15	40.63 (0.001)	0.063	22	0.880	0.762	0	1.596	0.976–2.610
Nausea and vomiting	8	450	396	2.602. (0.885–7.650)	0.00	3.305 (0.855)	0.082	0	0.711	0.756	3	1.880	0.752–4.702
Bloating/abdominal pain	24	1,274	1,250	1.464 (0.934–2.296)	71.06	79.47 (<0.001)	0.097	29	0.691	0.182	4	1.126	0.708–1.792
Borborygmus	4	418	391	0.964 (0.517–1.798)	55.37	6.72 (0.081)	0.908	0	0.497	0.292	0	0.964	0.517–1.798
Loose stools	4	418	391	0.695 (0.287–1.685)	85.16	20.21 (<0.001)	0.421	0	0.497	0.672	0	0.695	0.287–1.685
Rash	4	418	391	0.081 (0.019–0.342)	0.00	0.17 (0.981)	**0.001**	9	0.174	0.017	0	0.081	0.019–0.342

*Bold values: *P* <0.05. CI, confidence intervals; RRs, risk ratio; SMD, standard mean differences*.

#### Treatment Adherence

No difference was found between TCM alone and WM groups in both overall adherence, full adherence, and partial adherence rates (all *P-*values > 0.05; Table 2).

#### Adverse Drug Reactions

No group differences were found in most of the ADRs (e.g., diarrhea, nausea and vomiting, bloating/abdominal pain, borborygmus, and loose stools) (all *P*-values > 0.05; Table 2), while rash was less frequent (RR = 0.081, 95% CI: 0.019–0.342; *P* = 0.001; *I*^2^ = 0.0) in the TCM alone group compared to the WM group (Table 2).

Three RCTs compared relapse or exacerbation rates of constipation after discontinuation and all studies found that those receiving WM has a higher relapse rate than those receiving TCM. Specifically, one RCT found that the TCM group had a significantly lower relapse rate than the WM group at 1, 3, and 6 months after discontinuation (36). Another RCT had a similar finding (TCM: 13.24% vs. WM:36.37%; *X*^2^ = 8.45, *P* < 0.01) at 1 month after discontinuation (43). Jiang et al. (45) reported that some participants had relapsed after discontinuation in the WM group, but the result in the TCM group was not reported.

### Subgroup and Meta-Regression Analyses

For the moderate response rate, subgroup and meta-regression analyses found that diagnostic criteria of psychiatric disorders (CCMD-2/CCMD-2-R vs. CCMD-3 vs. ICD-10), geographical region (east vs. middle vs. west), analysis method (intent to treat vs. per-protocol), inpatient group (Yes vs. Mix), trial duration (β = −0.002, *P* = 0.128, *n* = 44 arms), total sample size (β = −0.0002, *P* = 0.473), and sample size in the TCM group (β = 0.0004, *P* = 0.315) and WM group (β = 0.0002, *P* = 0.717) did not moderate the primary results (all *P*-values > 0.05, Table 3), except for the publication year (β = −0.007, *P* = 0.0007) and Jadad score (β = 0.067, *P* < 0.001).

**Table 3:** Subgroup analyses of response rate and remission of traditional Chinese medicine compared with Western medicine for constipation.

**Subgroups**	**Categories** ** (number of studies)**	**Sample size**	**RRs**	**95% Confidence interval (%)** ** (lower, upper)**	***I^**2**^* (%)**	***P* within** ** subgroup**	***P* across** ** subgroups**
**Moderate response rate**							
Diagnostic criteria	CCMD-2/2-R (9)	918	1.214	(0.978, 1.509)	70.0	0.001	0.470
	CCMD-3 (27)	2,301	1.084	(1.012, 1.162)	75.1	<0.001	
	ICD-10 (1)	96	1.176	(0.989, 1.397)	0.0	1.000	
Analysis	ITT (47)	4,595	1.169	(1.097, 1.246)	78.0	<0.001	0.475
	PP (2)	194	1.112	(0.983, 1.257)	0.0	0.368	
Region	East (38)	3,873	1.136	(1.067, 1.208)	71.9	<0.001	0.118
	Middle (9)	786	1.465	(1.157, 1.856)	89.4	<0.001	
	West (2)	130	1.120	(0.946, 1.326)	12.7	0.284	
Inpatient	Yes (47)	4,575	1.172	(1.097, 1.251)	77.9	<0.001	0.064
	Mix (1)	134	1.066	(0.989, 1.149)	0.0	1.000	
Publication year*	≤ 2,008 (25)	2,788	1.152	(1.062, 1.251)	78.3	<0.001	0.627
	>2,008 (24)	2,293	1.189	(1.079, 1.310)	76.9	<0.001	
**Remission rate**							
Diagnostic criteria	CCMD-2 (7)	798	1.168	(0.883, 1.544)	74.2	0.001	0.818
	CCMD-3 (19)	1,536	1.212	(1.039, 1.414)	67.0	<0.001	
Analysis	ITT (30)	3,124	1.386	(1.185, 1.622)	79.6	<0.001	0.487
	PP (1)	98	1.083	(0.550, 2.133)	0.0	1.000	
Region	East (24)	2,672	1.219	(1.044, 1.423)	71.9	<0.001	**0.003**
	Middle (5)	383	3.713	(1.988, 6.902)	68.6	0.013	
	West (2)	167	1.191	(0.803, 1.767)	74.1	0.049	
Inpatient	Yes (28)	2,854	1.425	(1.185, 1.713)	80.2	<0.001	**0.035**
	Mix (2)	231	1.078	(0.898, 1.294)	42.7	0.186	
Publication year*	≤ 2,011 (16)	2,049	1.310	(1.074, 1.598)	77.7	<0.001	0.478
	>2,011 (15)	1,173	1.475	(1.137, 1.914)	81.0	<0.001	

*Based on the median splitting method.

*Bold values: *P* < 0.05. CCMD-2, Chinese Mental Disorder Classification and Diagnosis, Second Edition; CCMD-2-R, Chinese Mental Disorder Classification and Diagnosis, Second Edition, Revised; CCMD-3, Chinese Mental Disorder Classification and Diagnosis, Third Edition; ICD-10, International Classification of diseases, Tenth Edition; ITT, Intention to treat analysis; PP, per-protocol analysis*.

For the remission rate, subgroup analyses revealed that geographical region (*P* = 0.003) and inpatient group (*P* = 0.035) were significantly associated with the results (Table 3). Meta-regression analyses did not reveal significant moderating effects of the publication year (β = 0.009, *P* = 0.110), Jadad score (β = −0.036, *P* = 0.624), total sample size (β = 0.0007, *P* = 0.337), and sample size in the TCM (β = 0.001, *P* = 0.469) and WM groups (β = 0.002, *P* = 0.248) on the results, except for the trial duration (β = 0.009, *P* = 0.013, *n* = 23 arms).

### Sensitivity Analysis and Publication Bias

After excluding one outlying study (37) with two arms in which two WMs were used, the primary results did not significantly change (moderate response rate: RR = 1.156, 95% CI: 1.087–1.230, *P* < 0.001, *I*^2^ = 77.47%; marked response rate: RR = 1.391, 95% CI: 1.229–1.575, *P* < 0.001, *I*^2^ = 80.96%). In addition, we excluded each study one by one, and no significant changes were found in the moderate response rate, marked response rate, or remission rate (Supplementary Figures 8–10).

Both Egger's and Begg's-tests (all *P*-values > 0.05) and funnel plot did not detect publication bias in most outcomes, but publication bias was found in moderate response rate (Egger's-test: *t* = 4.248, *P* < 0.001; Begg's-test: *Z* = 2.793, *P* = 0.005; Table 2 and Supplementary Figure 5), marked response rate (Begg's-test: *Z* = 4.379, *P* <0.001; Egger's-test: *t* = 5.790, *P* < 0.001; Table 2 and Supplementary Figure 6), remission rate (Begg's-test: *Z* = 3.384, *P* <0.001; Egger's-test: *t* = 3.855, *P* < 0.001; Table 2 and Supplementary Figure 7), and rash (Egger's test, *P* = 0.017, Table 2). Duval and Tweedie's trim-and-fill analysis did not find any missing study, which indicates that no missing effect size qualitatively influence the primary results in all outcomes, except for the moderate response rate (missing studies = 8; new RR = 1.1, 95% CI: 1.030–1.174), marked response rate (missing studies = 10; new RR = 1.219, 95% CI: 1.067–1.392), remission rate (missing studies = 2; new RR = 1.440, 95% CI: 1.231–1.685), time of onset (missing studies = 1; new SMD = −0.028, 95% CI: −0.592–0.536), nausea and vomiting (missing studies = 3; new RR = 1.880, 95% CI: 0.752–4.702), and bloating/abdominal (missing studies = 4; new RR = 1.126, 95% CI: 0.708–1.792).

## Discussion

This was the first systematic review and meta-analysis that examined the efficacy and safety of TCM in treating antipsychotic-related constipation. Commonly prescribed TCM included Senna, Apricot Seed and Linum Formula, Ma Ren Wan, etc., while WM included Phenolphthalein, Glycerine Enema, etc. We found that TCM alone was superior to WM in terms of moderate response rate, marked response rate, and remission rate for constipation, while TCM alone was significantly associated with lower risk of rash. Skin rash is a common side effect associated with certain Western drug allergy (63) including antipsychotic drugs (64–66). In this meta-analysis compared to WM, TCM has a lower risk of rash. Traditional Chinese herbal medicine has been widely prescribed in China in treating antipsychotic drug-induced constipation (67), and TCM prescriptions strictly follow relevant treatment guidelines and regulations (68).

Our efficacy findings are similar to the findings of large case–control studies (69). An earlier review found that TCM was more effective than cisapride (RR = 0.24, 95% CI: 0.17–0.34), polyethylene glycol (RR = 0.14, 95% CI: 0.06–0.34), mosapride (RR = 0.33, 95% CI: 0.23–0.46), and phenolphthalein (RR = 0.24, 95% CI: 0.13–0.46) in treating functional constipation (13), which is consistent with the findings of this study and another meta-analysis (70). Traditional Chinese herbal medicine appears more effective for constipation than WM; however, due to the variety of components found across TCM, the mechanisms are still not clear. To date, no basic science research on the efficacy of TCM for constipation have been published.

Subgroup analyses revealed that the remission rate for treating constipation was moderated by geographical regions. When comparing TCM with WM, the RR of TCM vs. WM was 1.219 (95% CI: 1.044–1.423) in the eastern region and 3.713 (95% CI: 1.988–6.902) in the central region, while no difference was found in the western region of China. It should be noted that most studies were conducted in the eastern region, and only two studies with small sample size were conducted in the western region of China; therefore, the results of this subgroup analysis may not be stable. The different dietary habits among populations between regions in China may be partly responsible for the discrepancy. For example, many people in the central region of China (e.g., Hunan, Hubei, and Jianxi provinces) prefer spicy foods, which could increase the risk of constipation (71), while those in the eastern region prefer bland foods. The advantage of TCM in terms of remission rate was more obvious in the inpatient group compared to the mixed inpatient and outpatient group, which may be related to better treatment adherence among inpatients (72, 73) or due to a small number of studies on mixed patient sample (*n* = 2). As expected, meta-regression analysis found that a longer trial duration (β = 0.009, *P* = 0.013) was associated with a higher remission rate of constipation, probably because the delivery of TCM is more stable in longer studies. Meta-regression demonstrated that the moderate response rate was negatively related to the publication year (β = −0.007, *P* = 0.0007). We speculate that first-generation antipsychotics (FGAs) were widely used in the past, which often led to severe constipation (1). In the past decade, however, FGAs have been gradually replaced by second-generation antipsychotics (SGAs). In contrast, SGAs are less likely to cause severe constipation (74, 75). Unexpectedly, compared to those with only mild constipation, patients with severe constipation were often more likely to respond to TCM. We speculate that the doses of TCM and types of constipation may moderate this association although relevant data were insufficient to clarify this finding, which needs to be confirmed in future studies. The association of the higher response rate with higher-quality studies might be due to the fact that response is more likely to be identified in higher-quality studies, e.g., those with well-trained researchers and sensitive assessment tools.

The strengths of this systematic review and meta-analysis included the inclusion of both international and Chinese databases, large number of included studies, large sample size, and use of sophisticated analyses (e.g., subgroup, meta-regression, and sensitivity analyses). Some methodological limitations should be noted. First, all studies were conducted in China, which may limit the generalizability of the findings to other parts of the world. Additionally, the included studies were not large-scale RCTs. Second, the active ingredients of TCM and their optimal doses for constipation were not analyzed due to insufficient data. Unlike WM, due to the varied ingredients in most TCM, no dosages were provided as they were only administered as tablets and/or capsules in clinical practice. Also, due to different components and forms of TCM between included RCTs, head-to-head comparisons of TCM could not be conducted in this meta-analysis. Third, some factors related to constipation, such as lifestyle, outdoor activities and physical exercise status of participants, types and doses of antipsychotic medications, and major physical conditions, were not reported in most of the included studies. Finally, the efficacy and side effects between different TCMs were not compared due to the small number of studies in each subgroup.

In conclusion, this meta-analysis found that the efficacy of TCM on antipsychotic-related constipation was greater compared to WM, but certain side effects of TCM, such as rash, were less frequent. Hence, TCM appears to be an effective and safe treatment for antipsychotic-related constipation in clinical practice. However, these findings will need to be confirmed in future high-quality studies.

## Data Availability Statement

The original contributions presented in the study are included in the article/Supplementary Material, further inquiries can be directed to the corresponding authors.

## Author Contributions

W-WR, Y-TX, and WZ: study design. W-WR, J-JY, HQ, and SS: data collection, analysis, and interpretation. W-WR, HQ, and Y-TX: drafting of the manuscript. LZ, GU, and CN: critical revision of the manuscript. All authors: approval of the final version for publication.

## Conflict of Interest

The authors declare that the research was conducted in the absence of any commercial or financial relationships that could be construed as a potential conflict of interest. The reviewer XW declared a shared affiliation, though no other collaboration, with several of the authors, HQ, SS, and LZ, to the handling Editor. The reviewer QL declared a shared affiliation, though no other collaboration, with several of the authors, HQ, SS, and LZ, to the handling Editor.
