# Supplementary material for: Efficacy and Safety of Traditional Chinese Herbal Medicine for Antipsychotic-Related Constipation: A Systematic Review and Meta-Analysis of Randomized Controlled Trials
Source: Front Psychiatry. 2021 Apr 29;12:610171. doi: 10.3389/fpsyt.2021.610171 (PMC8116596; doi:10.3389/fpsyt.2021.610171)
Supplement: Supplementary file 1 [file Data_Sheet_1.docx]

**Supplemental Figure 1: Risk of bias**

|  | ***Random sequence generation (selection bias)*** | ***Allocation concealment (selection bias)*** | ***Blinding of participants and personnel*** | ***Blinding of outcome assessment (Symptom reduction, response)*** | ***Incomplete outcome data addressed (attrition bias)*** | ***Selective reporting (reporting bias)*** | ***Other sources of bias*** |
| --- | --- | --- | --- | --- | --- | --- | --- |
| Zhao L. M. et al., 1993 | **+** | **?** | **+** | **+** | **+** | **+** | **?** |
| Ding Z. M., 1998 | **?** | **?** | **?** | **?** | **+** | **+** | **?** |
| Wang L. H. et al., 1998 | **?** | **?** | **?** | **?** | **+** | **+** | **?** |
| Liu Z.J., 2001 | **?** | **?** | **?** | **?** | **+** | **+** | **?** |
| Hu, C.R. et al. 2002 | **?** | **?** | **?** | **?** | **+** | **+** | **?** |
| Li, C.W., 2003 | **?** | **?** | **?** | **?** | **+** | **+** | **?** |
| Zhang Z. F., 2003 | **?** | **?** | **?** | **?** | **+** | **+** | **?** |
| Li. K. Q. et al., 2005 | **?** | **?** | **?** | **?** | **+** | **+** | **?** |
| Li X. Y. et al., 2005 | **?** | **?** | **?** | **?** | **+** | **+** | **?** |
| Sheng C.D. et al., 2006 | **-** | **?** | **?** | **?** | **+** | **+** | **?** |
| Meng Q. L. et al., 2007 | **?** | **?** | **?** | **?** | **+** | **+** | **?** |
| Wang Z. F. et al., 2007 | **?** | **?** | **?** | **?** | **+** | **+** | **?** |
| Du Y. H. et al., 2008 | **?** | **?** | **?** | **?** | **+** | **+** | **?** |
| Han Y. D. et al., 2008 | **+** | **?** | **+** | **+** | **+** | **+** | **?** |
| Li. H. et al., 2008 | **-** | **?** | **?** | **?** | **+** | **+** | **?** |
| Xie Z. Y. et al., 2008 | **-** | **?** | **?** | **?** | **+** | **+** | **?** |
| Jiang X.J., 2009 | **?** | **?** | **?** | **?** | **+** | **+** | **?** |
| Liu B. Z. et al., 2010 | **?** | **?** | **?** | **?** | **+** | **+** | **?** |
| Li Z. Y., 2011 | **?** | **?** | **?** | **?** | **+** | **+** | **?** |
| Pan H. P. et al., 2012 | **?** | **?** | **?** | **?** | **+** | **+** | **?** |
| Wang Z. L. et al., 2013 | **?** | **?** | **?** | **?** | **+** | **+** | **?** |
| Chen X. et al., 2014 | **?** | **?** | **?** | **?** | **+** | **+** | **?** |
| Tian J.B. et al., 2014 | **?** | **?** | **?** | **?** | **+** | **+** | **?** |
| Han Z. M., 2015 | **+** | **?** | **?** | **?** | **+** | **+** | **?** |
| Ye F. Z. et al., 2016 | **-** | **?** | **?** | **?** | **+** | **+** | **?** |
| Zhao J. T. et al., 2016 | **?** | **?** | **?** | **?** | **+** | **+** | **?** |
| Tang Y., 2018 | **-** | **?** | **?** | **?** | **+** | **+** | **?** |
| Wang Z. et al., 2019 | **?** | **?** | **?** | **?** | **-** | **+** | **?** |
| Zhu Y., 2019 | **+** | **?** | **?** | **?** | **+** | **+** | **?** |
| Wu X H., 2020 | **+** | **?** | **?** | **?** | **+** | **+** | **?** |

+ : Low risk of bias, - : High risk of bias, ? : Unclear risk of bias, nd : not determined

*1. Random sequence generation (selection bias)*

*2. Allocation concealment (selection bias)*

*3. Blinding of participants and personnel*

*4. Blinding of outcome assessment (Symptom reduction, response)*

*5. Incomplete outcome data addressed (attrition bias)*

*6. Selective reporting (reporting bias)*

*7. Other sources of bias*

**Supplemental Table 1.** GRADE Analyses: Traditional Chinese medicine for constipation

| **Primary and secondary outcome** | **Active arms (N)** | **Risk of bias** | **Inconsistency** | **Indirectness** | **Imprecision^d^** | **Publication bias** | **Large effect** | **Overall quality of evidence^a^** |
| --- | --- | --- | --- | --- | --- | --- | --- | --- |
| *Total response rate* | 52 | Serious^b^ | Serious^c^ | No | No | Detected^e^ | No | +/-/-/-; Very low |
| *Marked response rate* | 44 | Serious^b^ | Serious^c^ | No | No | Detected^e^ | No | +/-/-/-; Very Low |
| *Remission rate* | 31 | Serious^b^ | Serious^c^ | No | No | Detected^e^ | No | +/-/-/-; Very low |
| *Time of onset* | 5 | Serious^b^ | Serious^c^ | No | No | Undetected | No | +/+/-/-; Low |
| *Total adherence rate* | 4 | Serious^b^ | Serious^c^ | No | No | Undetected | No | +/+/-/-; Low |
| *Full adherence rate* | 4 | Serious^b^ | Serious^c^ | No | No | Undetected | No | +/+/-/-; Low |
| *Partial adherence rate* | 4 | Serious^b^ | No | No | No | Undetected | No | +/+/+/-; Moderate |
| *Diarrhea* | 18 | Serious^b^ | Serious^c^ | No | No | Undetected | No | +/+/-/-; Low |
| *Nausea and vomiting* | 8 | Serious^b^ | No | No | No | Undetected | No | +/+/+/-; Moderate |
| *Bloating/abdominal pain* | 24 | Serious^b^ | Serious^c^ | No | No | Undetected | No | +/+/-/-; Low |
| *Borborygmus* | 4 | Serious^b^ | Serious^c^ | No | No | Undetected | No | +/+/-/-; Low |
| *Loose stools* | 4 | Serious^b^ | Serious^c^ | No | No | Undetected | No | +/+/-/-; Low |
| *Rash* | 4 | Serious^b^ | No | No | No | Detected^e^ | Very large^f^ | +/+/+/+; high |
| ^a^GRADE Working Group grades of evidence: High quality=further research is very unlikely to change our confidence in the estimate of effect. Moderate quality=further research is likely to have an important impact on our confidence in the estimate of effect and may change the estimate. Low quality=further research is very likely to have an important impact on our confidence in the estimate of effect and is likely to change the estimate. Very low quality=we are very uncertain about the estimate.  ^b^ Pooled data from studies with single blind or open label.  ^c^ Meta-analytic results presented a serious inconsistency when I^2^ values were greater than 50% or P<0.1 in the *Q* statistics.  ^d^ For continuous outcomes, N<400; For dichotomous outcomes, N<300.  ^e^ Meta-analytic results presented the publication bias by Egger or/and Begg tests.  ^f^ Studies with large effects provided increased quality of evidence. Large effects = 2<RR<0.5 or standard mean differences < -0.6. Very large effects=5<RR<0.2 or standard mean differences < -0.8.  Abbrevations: GRADE=grading of recommendations assessment, development, and evaluation. | | | | | | | | |

**Supplemental Table 2.** Search Strategy: Pubmed and CNKI

**PubMed**

("Dyschezia" OR "Colonic Inertia" OR "constipat*" OR "obstipation" OR "laxatives" OR "Intestinal obstruction" OR "bowel obstruction" OR "Ileus" OR "paralytic ileus" OR "anticholinergic (side) effect" OR "antimuscarinic (side) effect") AND ("Traditional Chinese Medicine" OR "Chinese Traditional Medicine" OR "Chinese traditional" OR "Chinese medicine" OR "Chinese Herbal Drugs" OR "Chinese herbal medicine" OR "Chinese Drugs, Plant" OR "Medicine, Traditional" OR Ethnopharmacology OR Ethnomedicine OR Ethnobotany OR "Medicine, Kampo" OR Kanpo OR TCM OR TCD OR Phytotherapy OR Herbology OR "Plants, Medicinal" OR "Plant Preparation" OR "Plant Extract" OR "Plants, Medicine" OR "Materia Medica" OR "Single Prescription" OR Herbs OR "Chinese Medicine Herb" OR "Herbal Medicine" OR "integrative medicine" OR "alternative medicine") AND ("randomized controlled trial" OR "controlled clinical trial" OR random* OR placebo OR "drug therapy" OR control)

**CNKI**

SU=('精神疾病' + '抗精神病药' ) * ('便秘' + '排便异常' + '脾胃病' + '大肠热结'+ '胃中燥矢'+ '排便困难' ) * ('中医' + '中药' + '中医疗法' + '辨病论治' + '辨证' + '辨证论治' + '辨症' + '辩证' + '汉方' + '祖国医学' + '传统医学' + '传统治疗' + '传统疗法' + '替代医学' + '替代治疗' + '中国传统医学' + '民族医药' + '民族医学' + '草药' + '中草药' + '中药疗法' + '中西药' + '传统医药' + '中成药' + '植物药' + '中医治法') * ('随机' + '对照' + '安慰剂')

**Supplemental Table 3.** Criteria for clinical efficacy of studies included in this meta-analysis

| No. | Criteria for clinical efficacy |
| --- | --- |
| 1 | Within 24 hours after taking the medicine:  Response: defecation  Moderate response=response |
| 2 | Remission: without constipation in 72 consecutive hours  Moderate response=remission;  Marked response=remission |
| 3 | Within 24 hours after taking the medicine:  Response: defecation  Moderate response=response |
| 4 | Response: recovery of defecation habits and ease of defecation; or reduction of constipation days  Moderate response=response |
| 5 | After taking the medicine:  Response: defecation volume is from 100 g to 500 g  Marked response: defecation volume is more than 500 g  Moderate response=response + marked response  Marked response=marked response |
| 6 | Remission: without constipation in 72 consecutive hours  Moderate response=remission;  Marked response=remission |
| 7 | Remission: without constipation in 72 consecutive hours  Moderate response=remission;  Marked response=remission |
| 8 | Response: defecation within 3 days, moist stool, poor bowel movements  Remsision: defecation once within 2 days; moist stool and smooth bowel movements, no recurrence in a short time  Moderate response=response + remission  Marked response =remission |
| 9 | Within 24 hours after taking the medicine:  Response: defecation volume is from 100 g to 500 g  Marked response: defecation volume is more than 500 g  Moderate response=response + marked response  Marked response=marked response |
| 10 | Within 10 hours after taking the medicine:  Marked response: defecation  Moderate response=marked response  Marked response=marked response |
| 11 | Response: reduced defecation interval time by 24 h or improved symptoms of constipation, and all other symptoms improved  Marked response: significantly alleviate constipation, defecation interval time and quality will be near normal or slightly dry stool and defecation interval time during 72 h, most of the other symptoms disappear  Remission: normal stool or return to the pre-ill level, and some symptoms (abdominal pain, bloating, anal fissure bleeding, dry mouth and bitterness) disappear  Moderate response=response + marked response + remission  Marked response=marked response+remission |
| 12 | After taking the medicine:  Response: within 24 to 48 hours, the stool is smooth  Marked response: within 12-24 hours, breathing and bowel movements are smooth  Moderate response=response + marked response  Marked response=marked response |
| 13 | Response: reduced defecation interval time by 24 h or improved symptoms of constipation, and all other symptoms improved  Marked response: significantly alleviate constipation, defecation interval time and quality will be near normal or slightly dry stool and defecation interval time during 72 h, most of the other symptoms disappear  Remission: normal stool or return to the pre-ill level, and some symptoms (abdominal pain, bloating, anal fissure bleeding, dry mouth and bitterness) disappear  Moderate response=response + marked response + remission  Marked response=marked response+remission |
| 14 | Response: defecation within 24 hours after medication  Moderate response=response |
| 15 | After treatment:  Response: the degree of constipation is from other level to level two  Marked response: the degree of constipation is from other level to level one Remission: the degree of constipation is from other level to level zero  Moderate response=response + marked response + remission  Marked response=marked response+remission |
| 16 | Response: defecation within 24 hours after medication  Moderate response=response |
| 17 | Response: defecation within 3 days, moist stool, poor bowel movements  Remission: disappearance of the main symptoms, defecation once within 2 days, moist stool and smooth bowel movements, no recurrence in a short time  Moderate response=response + remission  Marked response=remission |
| 18 | Response: incompletely defecation within 12-24 hours  Marked response: incompletely defecation within 6- 12 hours  Remission: thoroughly defecation within 6 hours  Moderate response=response + marked response + remission  Marked response=marked response+remission |
| 19 | Response: the dry stool has improved, the interval between stools is shortened by 1-2 days, and other symptoms have improved  Marked response: significantly improvement of constipation, almost normal of stool quality, stool interval of 1 to 2 days, disappearance of two thirds of other symptoms  Remission: stools softened, defecation once a day, other symptoms (e.g. abdominal fullness, abdominal pain etc.) disappeared  Moderate response=response + marked response + remission  Marked response=marked response+remission |
| 20 | Remission: constipation disappears completely  Moderate response=remission  Marked response=remission |
| 21 | Remission: without constipation in 72 consecutive hours  Moderate response=remission  Marked response=remission |
| 22 | Remission: without constipation in 72 consecutive hours  Moderate response=remission  Marked response=remission |
| 23 | Response: the dry stool has improved, the interval between stools is shortened by 1-2 days, and defecation less than twice a week  Marked response: significantly improvement of constipation, almost normal of stool quality, defecation twice a week at least  Remission: stools softened, defecation 3 times a week or more, other subjective symptoms (e.g. abdominal fullness, abdominal pain etc.) disappeared  Moderate response=response + marked response + remission  Marked response=marked response+remission |
| 24 | Response: the dry stool has improved, the interval between stools is shortened by 1-2 days, and other symptoms have improved  Marked response: significantly improvement of constipation, almost normal of stool quality, stool interval of 1 to 2 days, disappearance of two thirds of other symptoms  Remission: stools softened, defecation once a day, other symptoms (e.g. abdominal fullness, abdominal pain etc.) disappeared  Moderate response=response + marked response + remission  Markedly response=marked response+remission |
| 25 | Response: after taking the medicine for 24 to 48 hours, the stool is smooth  Marked response: after taking the medicine for 12-24 hours, breathing and bowel movements are smooth  Moderate response=response + marked response  Marked response=marked response |
| 26 | Within 12 hours after taking the medicine:  Response: defecation volume is from 100 g to 500 g  Marked response: defecation volume is more than 500 g  Moderate response=response + marked response  Marked response=marked response |
| 27 | Response: slightly improved symptoms of constipation, defecation once within 3 days  Marked response: significantly alleviate constipation, slightly smooth defecation once within 2 days, slightly stools softened  Remission: Complete disappearance of constipation, stools softened, smooth defecation once a day  Moderate response=response + marked response + remission  Marked response=marked response+remission |
| 28 | After the treatment:  Response: defecation once within 2 days  Marked response: defecation once a day  Moderate response=response + marked response  Marked response=marked response |
| 29 | Remission: without constipation in 3 consecutive days  Moderate response=remission  Marked response=remission |
| 30 | Response: diarrhea symptom disappeared; stool turned to be moist, poor bowel movements  Remission: disappearance of constipation, normal defecation, strip stools with less water, smooth stools, no diarrhea symptoms after continuous use of antipsychotics for 2 weeks  Moderate response=response+remission;  Marked response=remission |

**
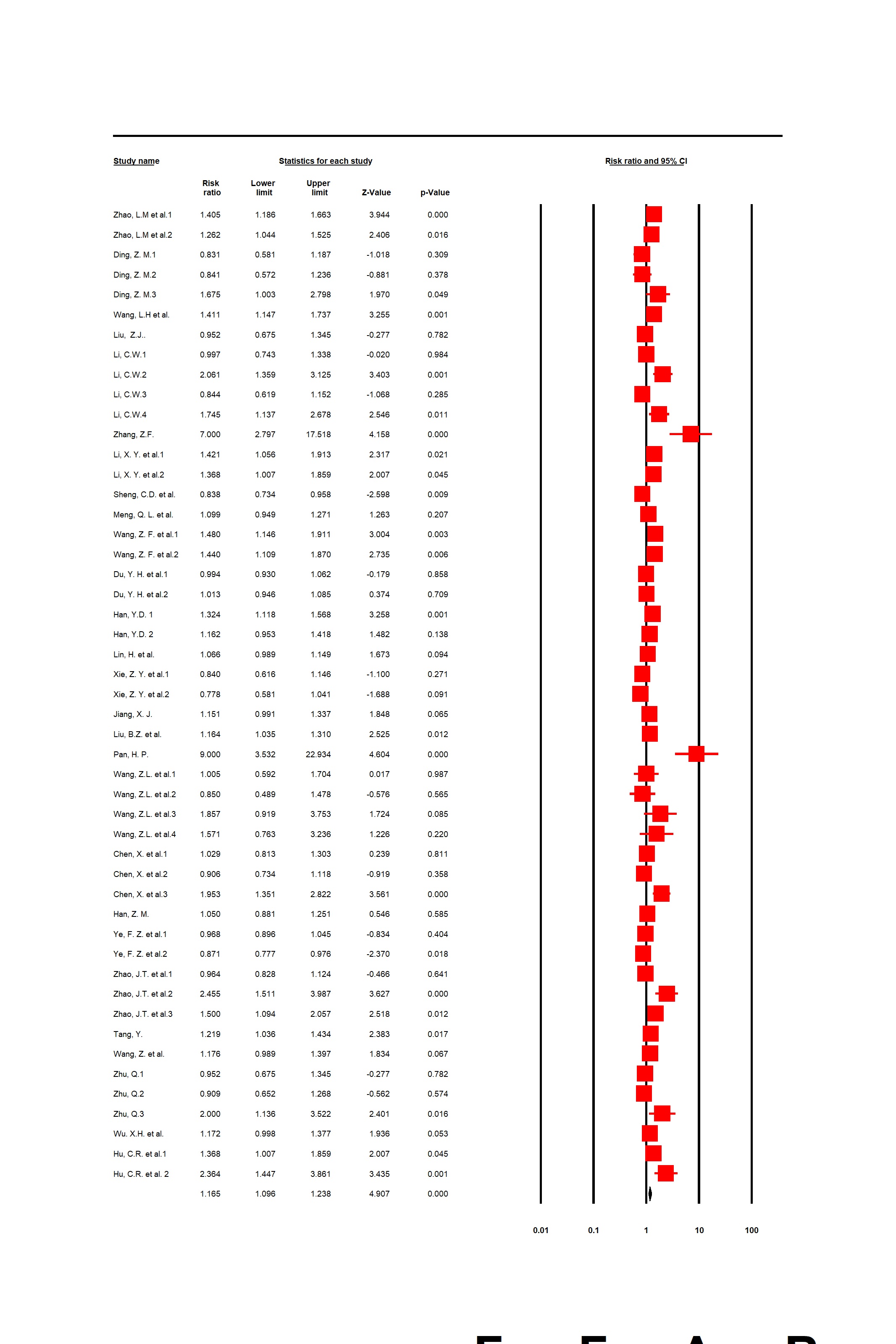
**

**Supplemental Figure 2. Traditional Chinese herbal medicine for antipsychotic-related constipation: Forest plot for moderate response rate**

**
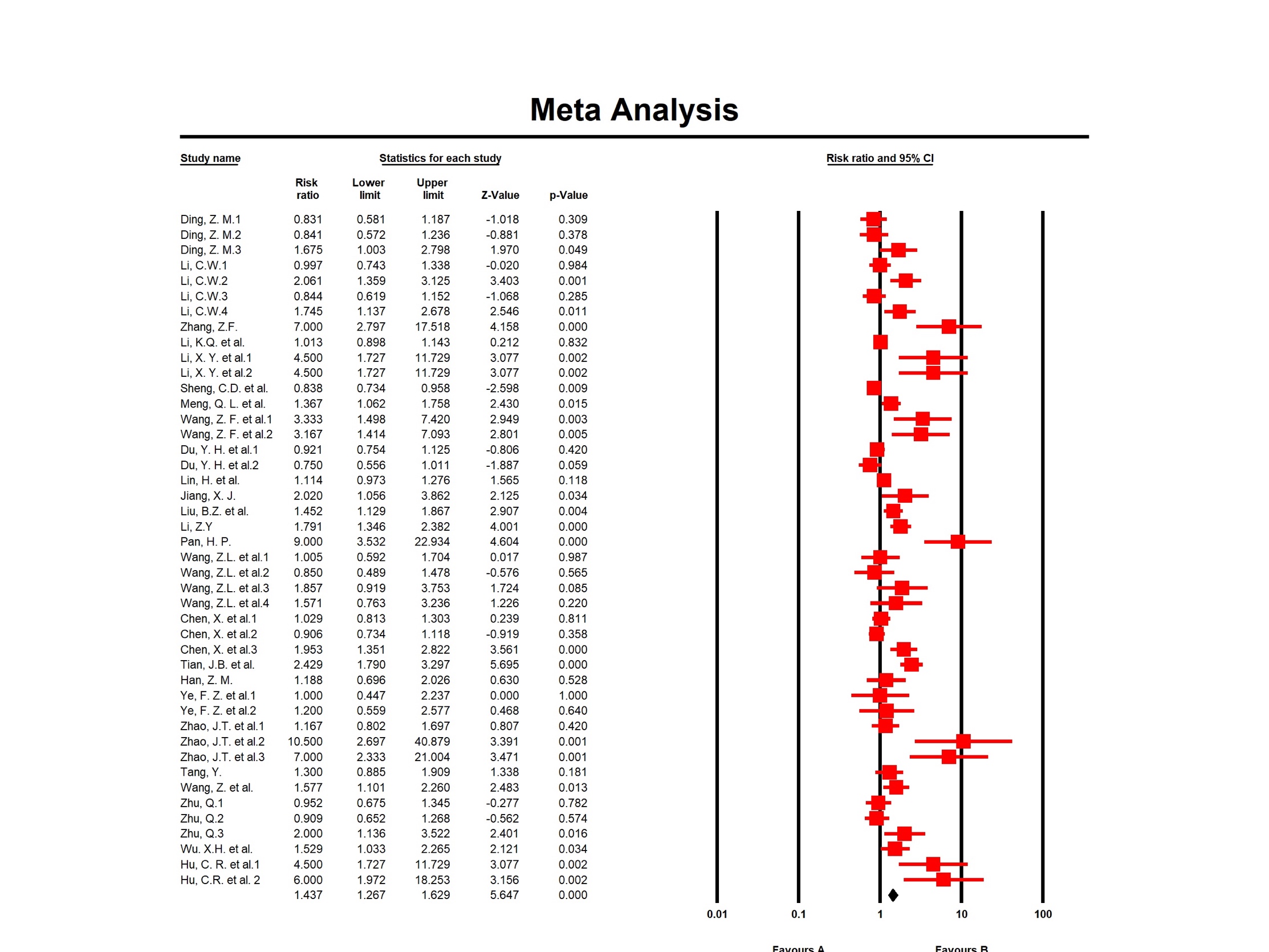
**

**Supplemental Figure 3. Traditional Chinese herbal medicine for antipsychotic-related constipation: Forest plot for marked response rate**

**
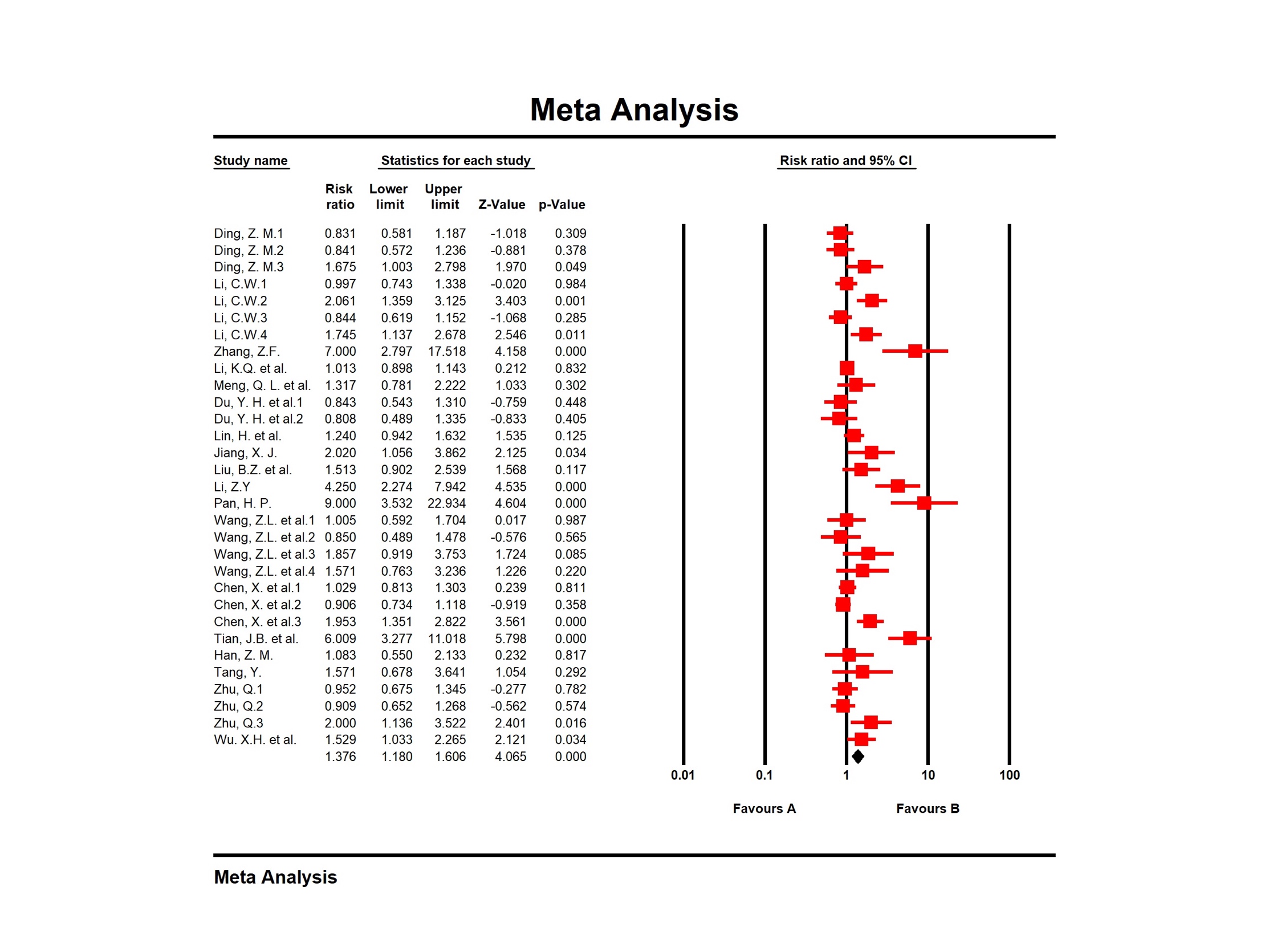
**

**Supplemental Figure 4. Traditional Chinese herbal medicine for antipsychotic-related constipation: Forest plot for remission rate**

**
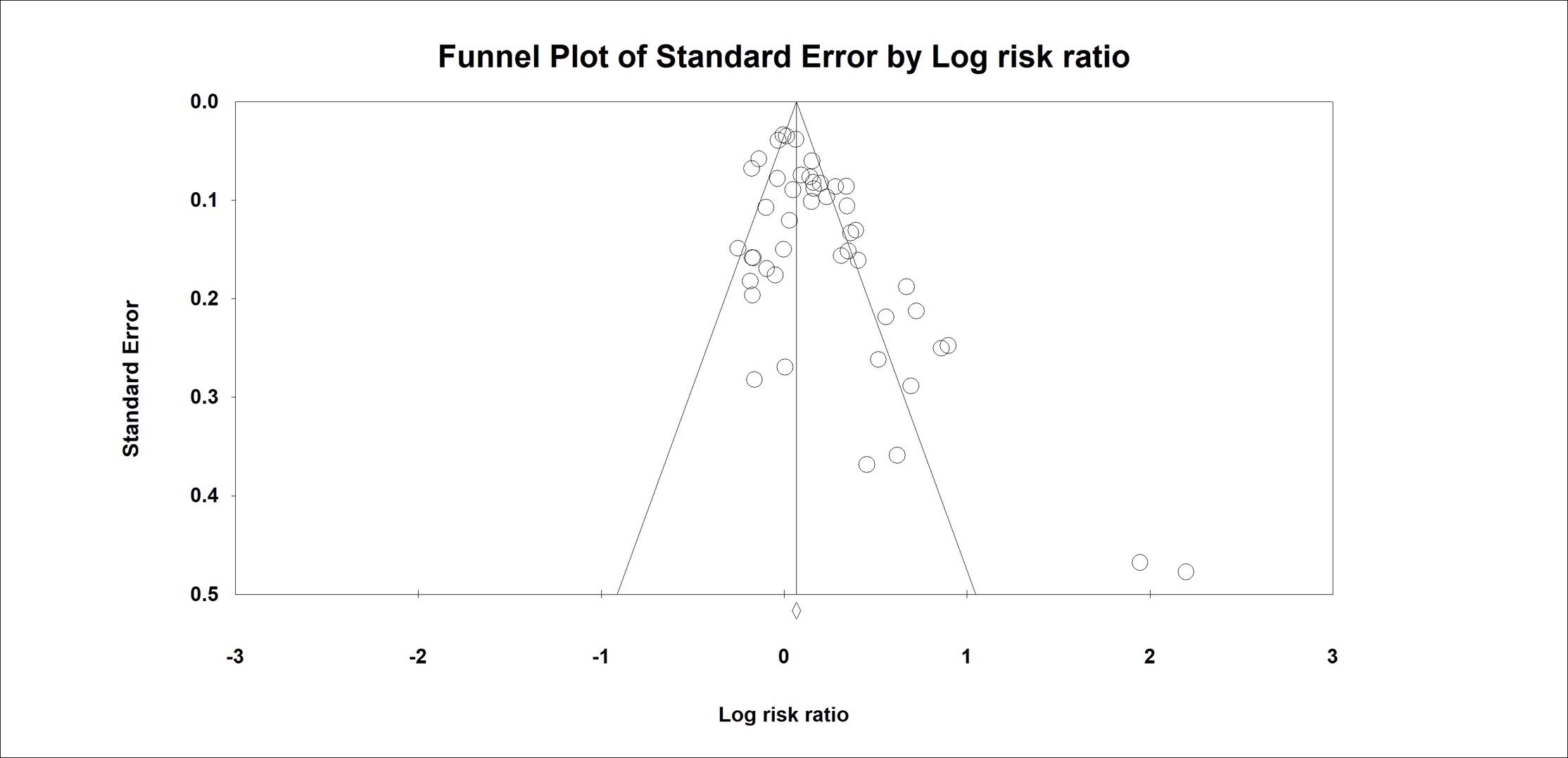
**

**Supplemental Figure 5. Traditional Chinese herbal medicine for antipsychotic-related constipation: Funnel plot of publication bias for moderate response rate**

**
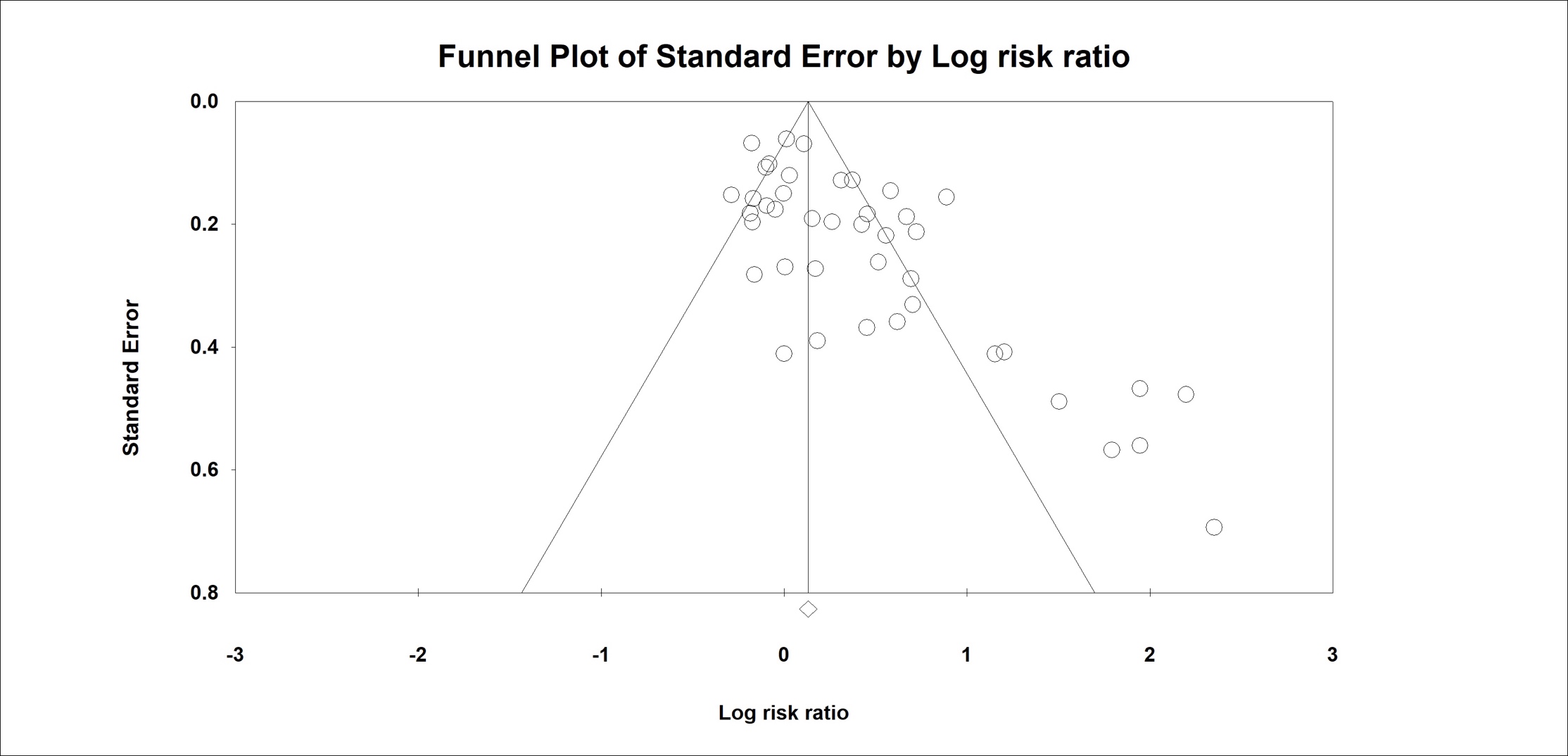
**

**Supplemental Figure 6. Traditional Chinese herbal medicine for antipsychotic-related constipation: Funnel plot of publication bias for marked response rate**

**
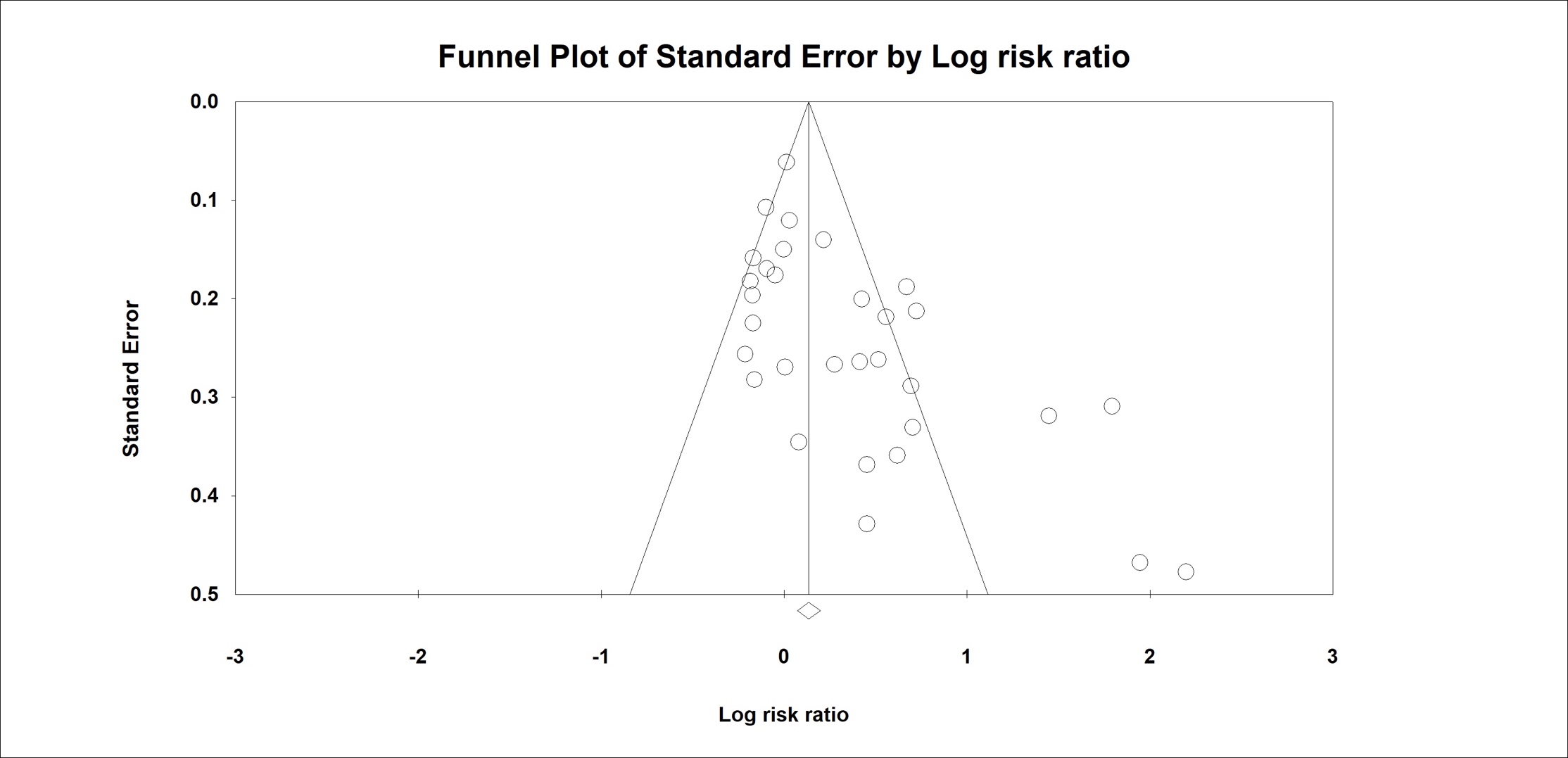
**

**Supplemental Figure 7. Traditional Chinese herbal medicine for antipsychotic-related constipation: Funnel plot of publication bias for remission rate**

**
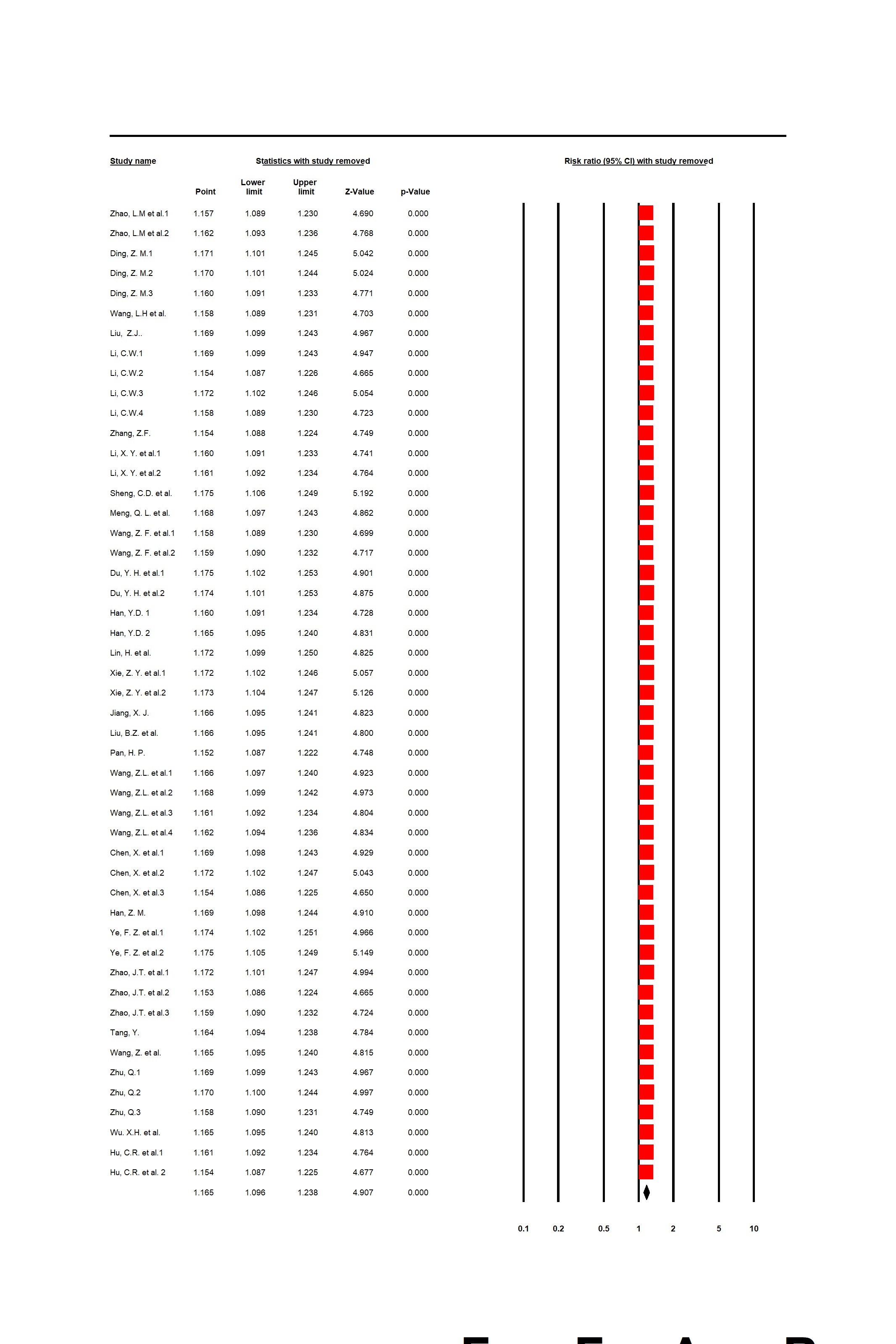
**

**Supplemental Figure 8. Traditional Chinese herbal medicine for antipsychotic-related constipation: Forest plot of sensitive analysis for moderate response rate**

**
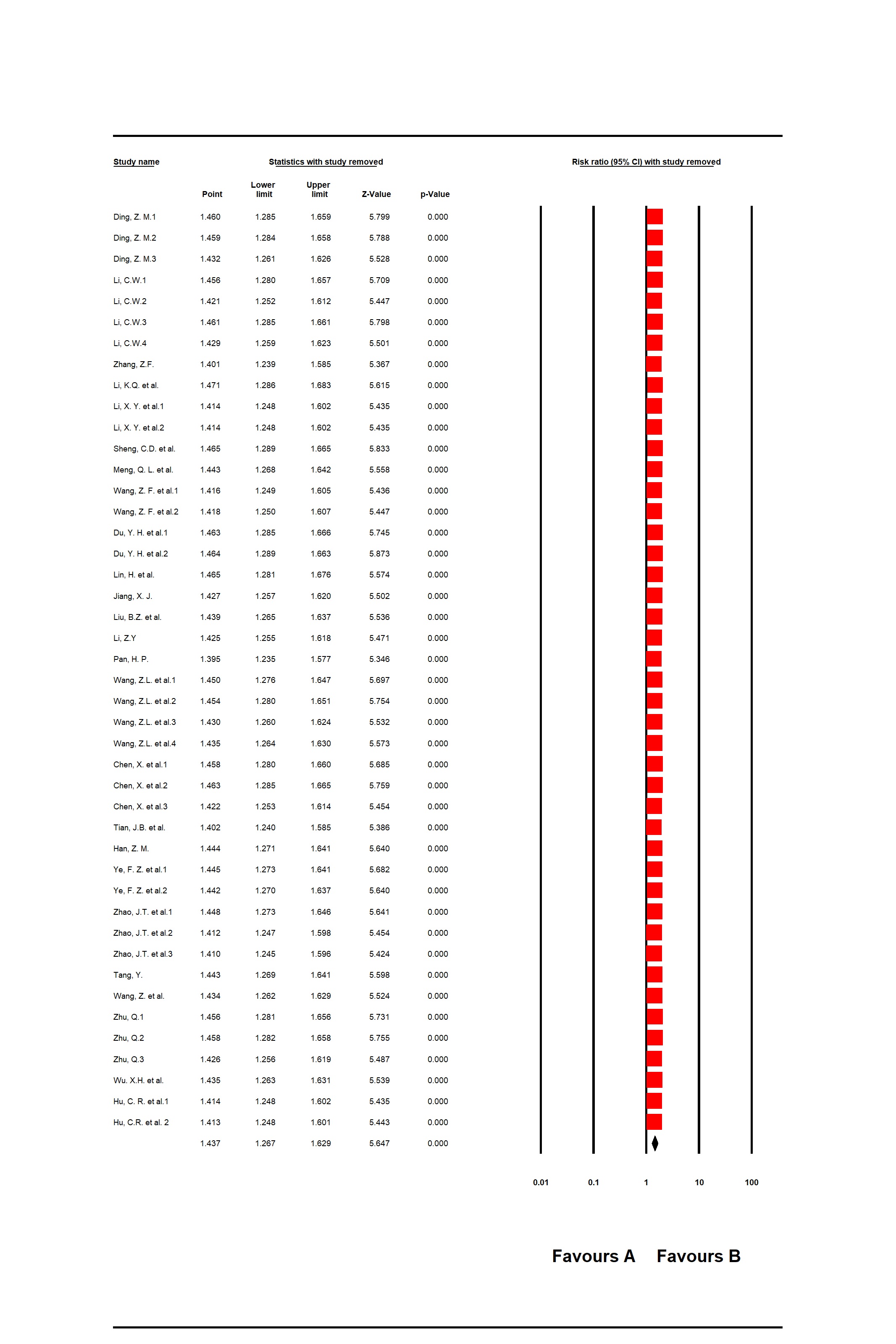
**

**Supplemental Figure 9. Traditional Chinese herbal medicine for antipsychotic-related constipation: Forest plot of sensitive analysis for marked response rate**

**
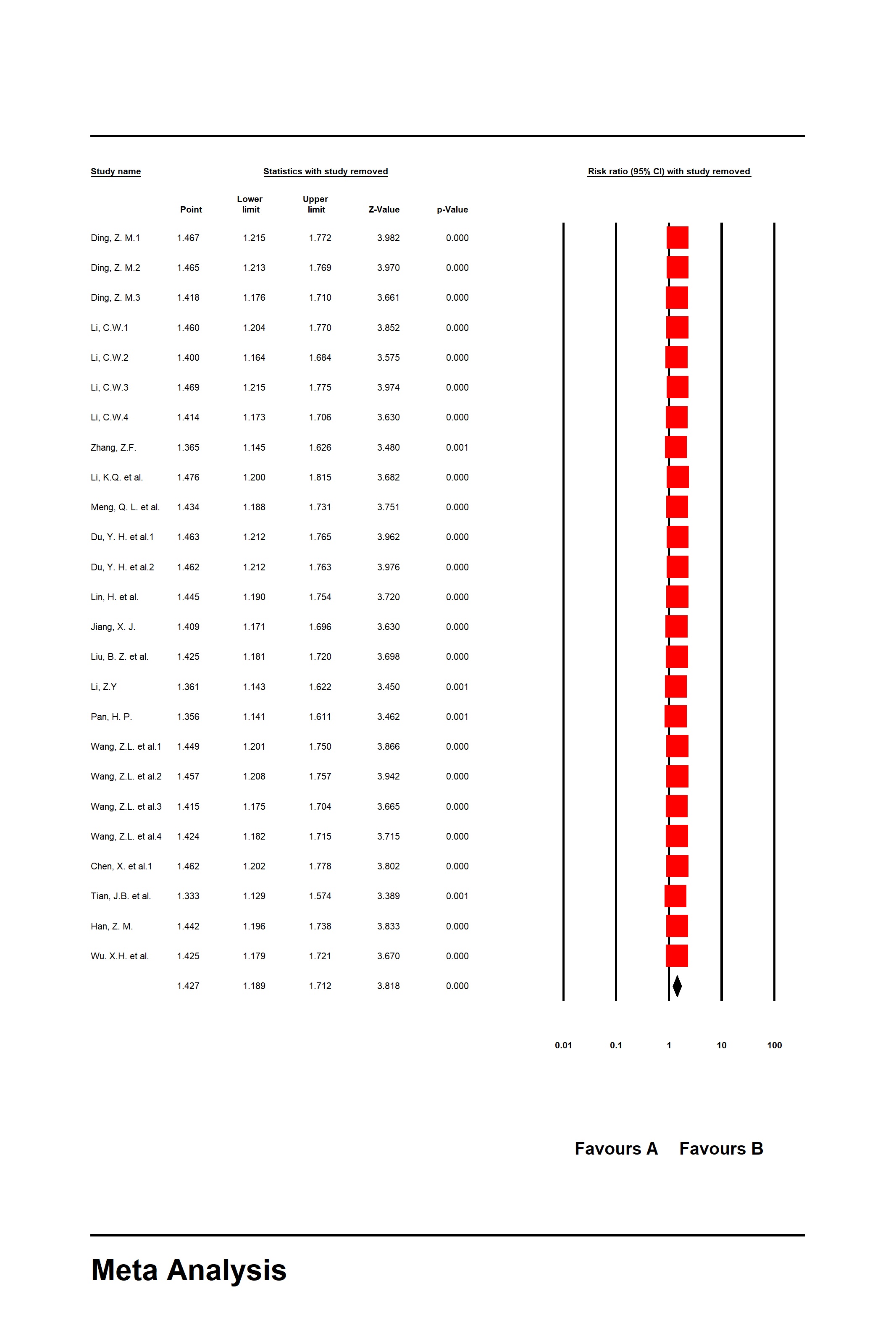
**

**Supplemental Figure 10. Traditional Chinese herbal medicine for antipsychotic-related constipation: Forest plot of sensitive analysis for remission rate**
